# Antibiotics Shaping Bacterial Genome: Deletion of an IS*91* Flanked Virulence Determinant upon Exposure to Subinhibitory Antibiotic Concentrations

**DOI:** 10.1371/journal.pone.0027606

**Published:** 2011-11-11

**Authors:** Laura Pedró, Rosa C. Baños, Sonia Aznar, Cristina Madrid, Carlos Balsalobre, Antonio Juárez

**Affiliations:** 1 Institut de Bioenginyeria de Catalunya, Parc Científic de Barcelona, Barcelona, Spain; 2 Departament de Microbiologia, Facultat de Biologia, Universitat de Barcelona, Barcelona, Spain; University of Groningen, The Netherlands

## Abstract

The nucleoid-associated proteins Hha and YdgT repress the expression of the toxin α-hemolysin. An *Escherichia coli* mutant lacking these proteins overexpresses the toxin α-hemolysin encoded in the multicopy recombinant plasmid pANN202-312R. Unexpectedly, we could observe that this mutant generated clones that no further produced hemolysin (Hly^-^). Generation of Hly^-^ clones was dependent upon the presence in the culture medium of the antibiotic kanamycin (km), a marker of the *hha* allele (*hha*::Tn*5*). Detailed analysis of different Hly^-^ clones evidenced that recombination between partial IS*91* sequences that flank the *hly* operon had occurred. A fluctuation test evidenced that the presence of km in the culture medium was underlying the generation of these clones. A decrease of the km concentration from 25 mg/l to 12.5 mg/l abolished the appearance of Hly^-^ derivatives. We considered as a working hypothesis that, when producing high levels of the toxin (combination of the *hha ydgT* mutations with the presence of the multicopy hemolytic plasmid pANN202-312R), the concentration of km of 25 mg/l resulted subinhibitory and stimulated the recombination between adjacent IS*91* flanking sequences. To further test this hypothesis, we analyzed the effect of subinhibitory km concentrations in the wild type *E. coli* strain MG1655 harboring the parental low copy number plasmid pHly152. At a km concentration of 5 mg/l, subinhibitory for strain MG1655 (pHly152), generation of Hly^-^ clones could be readily detected. Similar results were also obtained when, instead of km, ampicillin was used. IS*91* is flanking several virulence determinants in different enteric bacterial pathogenic strains from *E. coli* and *Shigella*. The results presented here evidence that stress generated by exposure to subinhibitory antibiotic concentrations may result in rearrangements of the bacterial genome. Whereas some of these rearrangements may be deleterious, others may generate genotypes with increased virulence, which may resume infection.

## Introduction

Pathogenic bacteria incorporate in their genomes DNA stretches that have been horizontally acquired (HGT DNA) and that contain genes required for the colonization of their hosts. The term “pathogenicity island” (PAI) refers to DNA regions that can be unstable, carry virulence determinants and are usually HGT [1], [2]. These DNA regions can also include, or be flanked by, insertion elements (IS elements) which, in turn, may facilitate integration in different regions of the chromosome. Genes other than those specifically required for virulence can be present in these islands (i.e., antibiotic resistance determinants, catabolic genes or even paralogues of genes encoded in the core genome). Several pathogenicity islands can be spontaneously excised from the chromosome at detectable rates. In most cases, the instability of PAIs is due to their precise excision from the chromosome via recombination between identical directed repeated sequences that flank the element. These short (9 to 20 bp) repeats are analogous to phage *att* sites. Upon PAI deletion, only one copy of the directed repeat remains on the chromosome [3]. In other instances, IS elements that flank some PAIs mediate deletion of the flanked DNA sequences [4]. As an example, recombination between two flanking IS*100* elements has been shown to account for deletion of the high-pathogenicity island (HPI) from *Yersinia pestis* [5].

IS*91* was first discovered in plasmids encoding the toxin α-hemolysin (Hly) in *Escherichia coli* [6]. IS*91* is usually associated to various plasmid and chromosomal pathogenicity islands that harbor the α-hemolysin operon [7], [8], [9], and it was suggested that this element was involved in the dissemination of these pathogenicity determinants [10]. Although initially considered a rarity among IS elements, new examples have evidenced this element flanking several virulence determinants [11]. In addition to the *hly* genes, IS*91* and closely related isoforms have also been located adjacent to several other virulence determinants in enteropathogenic, enterohemolytic and enterotoxigenic strains of *E. coli* [12], [13]. IS*91* like elements differ from other IS elements in that they lack terminal inverted repeats and are considered to transpose by a mechanism termed rolling circle transposition [14].

Bacterial cells have developed several strategies to cope with sudden changes in environmental conditions that result in stress. Several stress-responsive mechanisms rely on regulatory circuits that modify the gene expression pattern [15]. In addition to altering DNA expression, it is also known that stress may account for changes in the DNA sequences. The SOS response, triggered by the accumulation of ssDNA, is a well-characterized mechanism by which bacterial cells, in response to environmental factors that cause DNA damage, increase the mutation or DNA rearrangement rates [16]. Stress-induced adaptative amplifications of DNA have been reported in living organisms [17] and, within the bacterial kingdom, are most studied in *E.coli* [18], [19], [20]. This microorganism can respond to starvation stress by amplifying specific DNA sequences that allow cells to adjust to these conditions. The general stress response regulator RpoS appears to be required for that response [19]. In enteric bacteria deletions of large DNA stretches, such as those flanked by directed repeats or IS elements, are known to occur. It has been reported that these DNA rearrangements occur spontaneously, at fixed rates [21]. However, in the plant pathogen *Pseudomonas syringae* pv. *Phaseolicola*, stress generated by host defenses leads to excision of certain genomic islands and other DNA rearrangements [22]. In this work, experimental data that correlate environmental stress with induction of DNA deletions in Enterobacteria are presented. Our results let us to conclude that exposure to subinhibitory concentrations of certain antibiotics accounts for deletions of genomic islands flanked by partial IS*91* sequences.

## Results

### Kanamycin-dependent deletion of a DNA fragment including the operon encoding the toxin α-hemolysin in *Escherichia coli*

**Figure 1:**
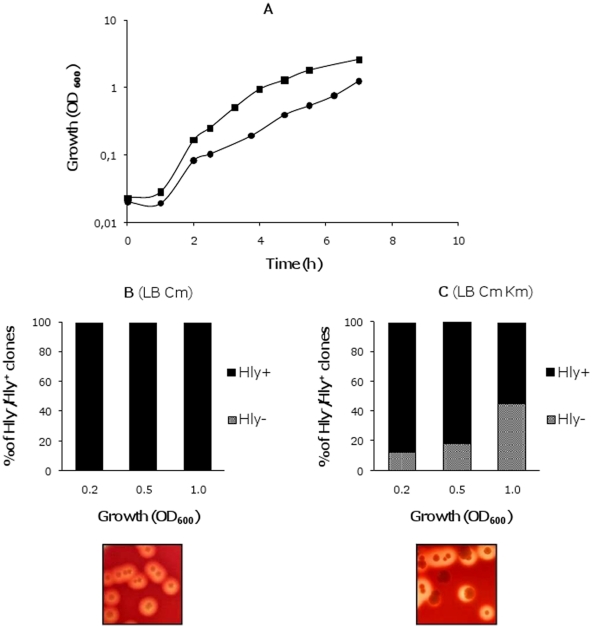
Generation of non-hemolytic colonies from strain MG1655HY (pANN202-312R). A. Growth curves of strain MG1655HY (pANN202-312R) in the absence (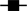) and presence (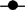) of km; B and C, proportion of hemolytic and non-hemolytic clones in both cultures at different stages of the growth curve. Inlets show blood agar plates inoculated with cells collected from both cultures.

Production of *E. coli* α-hemolysin is tightly regulated. The nucleoid-associated proteins H-NS and Hha interact to silence expression of the toxin under several environmental conditions, such as low temperature and high osmolarity [23]. Proteins of the Hha family mimic the oligomerization domain of H-NS and form complexes with this latter protein to repress the expression of several virulence determinants (as reviewed in [24]). *hns/hha* mutants upregulate hemolysin expression, which can be readily visualized on blood agar plates because of their large hemolysis haloes (see Figure 1B). In the *E. coli* chromosome paralogues to both H-NS and Hha proteins are found: the StpA and YdgT proteins respectively [25]. These protein paralogues can compensate for the lack of either H-NS or Hha, partially attenuating the mutant phenotype. Hence, double *hha ydgT* mutants show a higher derepression of the hemolysin expression than single *hha* mutants [26]. The characterization studies of a double *hha ydgT* mutant from the *E. coli* strain MG1655 (strain MG1655HY) raised an unexpected phenotype. When strain MG1655HY was transformed with the plasmid pANN202312R (a medium-copy plasmid containing a 17010 bp *Sal*I/*Sal*I fragment with the complete *hly* operon from the wt plasmid pHly152 [27]), non-hemolytic (Hly^-^) colonies could be detected on blood agar plates. Interestingly, detection of non-hemolytic derivatives was dependent upon the presence of kanamycin (km) (a marker of the *hha* mutation) at a concentration of 25 mg/l in the culture medium. We decided to characterize the non-hemolytic derivatives and their relationship to the presence of km in the medium. Overnight cultures of the strain MG1655HY (pANN202-312R) in LB containing chloramphenicol (cm, the plasmid marker) were used to inoculate (1∶100) fresh LB cm and LB cm km media. Growth was monitored and the presence of non-hemolytic clones was quantified. When compared to the growth rate in km-free LB medium, the growth rate in medium containing km was significantly reduced (Figure 1A). Moreover, in LB cm km cultures but not in cultures in LB cm, Hly^-^ clones could be detected. Their proportion increased at the later growth stages (Figures 1B and C).

### Hly^-^ clones from strain MG1655HY harbor pANN202-312R plasmid derivatives exhibiting two different deletion patterns

**Figure 2:**
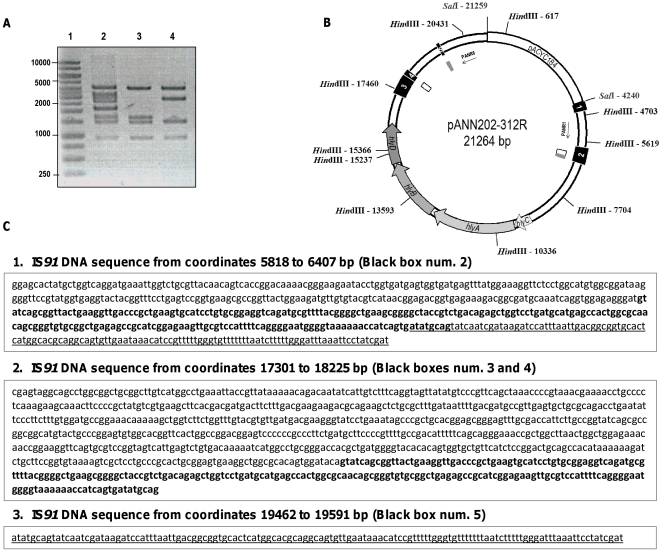
Mapping of the IS*91* directed repeats that generate the two deletion patterns observed. A. *Hin*dIII restriction analysis of pANN202-312R plasmid DNA (lane 2) and plasmid DNA isolated from two Hly^-^ clones exhibiting the two different deletion patterns identified (lanes 3 and 4, deletions 1 and 2 respectively). Lane 1 corresponds to the molecular mass marker. B. Physical map of plasmid pANN202-312R. *Hind*III and *Sal*I restriction sites and their corresponding coordinates are shown. Black boxes 1 to 5 correspond to the five partial IS*91* sequences that flank the *hly* genes. Internal grey boxes correspond to IS*91* direct repeats generating deletion 1. Internal white boxes correspond to IS*91* direct repeats generating deletion 2. C. DNA sequences of both IS*91* direct repeats that flank the deletions. Box 1 shows the DNA sequence corresponding to IS*91*-2 from coordinates 5818 to 6407. The direct repeat generating deletion 1 is shown underlined. The direct repeat generating deletion 2 is shown in bold. Box 2 corresponds to the DNA sequences of IS*91*-3 and 4. The direct repeat generating deletion 2 is shown in bold. Box 3 corresponds to the DNA sequence of IS*91*-5. The direct repeat generating deletion 1 is shown underlined.

To gain insight into the origin of the Hly^-^ clones, plasmid DNA from thirty Hly^-^ colonies isolated in 6 independent experiments and was characterized by restriction analysis. Interestingly, two deletion patterns were found (Figure 2A). The complete sequence of the 17010 bp *Sal*I/*Sal*I fragment of plasmid pANN202-312R was obtained (GenBank accession number BankIt1460463 Seq1 JN130365), and the sites affected in both types of deletions were precisely determined (Figure 2B internal boxes in white and grey). The DNA sequence obtained complemented previous hybridization studies [6] and evidenced that five incomplete IS*91* elements flank the hemolytic determinant of plasmid pHly152. Two DNA motifs, located in IS*91* incomplete elements 2, 4 and 5 are repeated in direct orientation flanking both ends of the *hly* genes (Figure 2C, Figure S1). These sequences are the targets for the deletions that generate the two different restriction patterns observed.

### Kanamycin accounts for the generation of Hly^-^ clones

**Figure 3:**
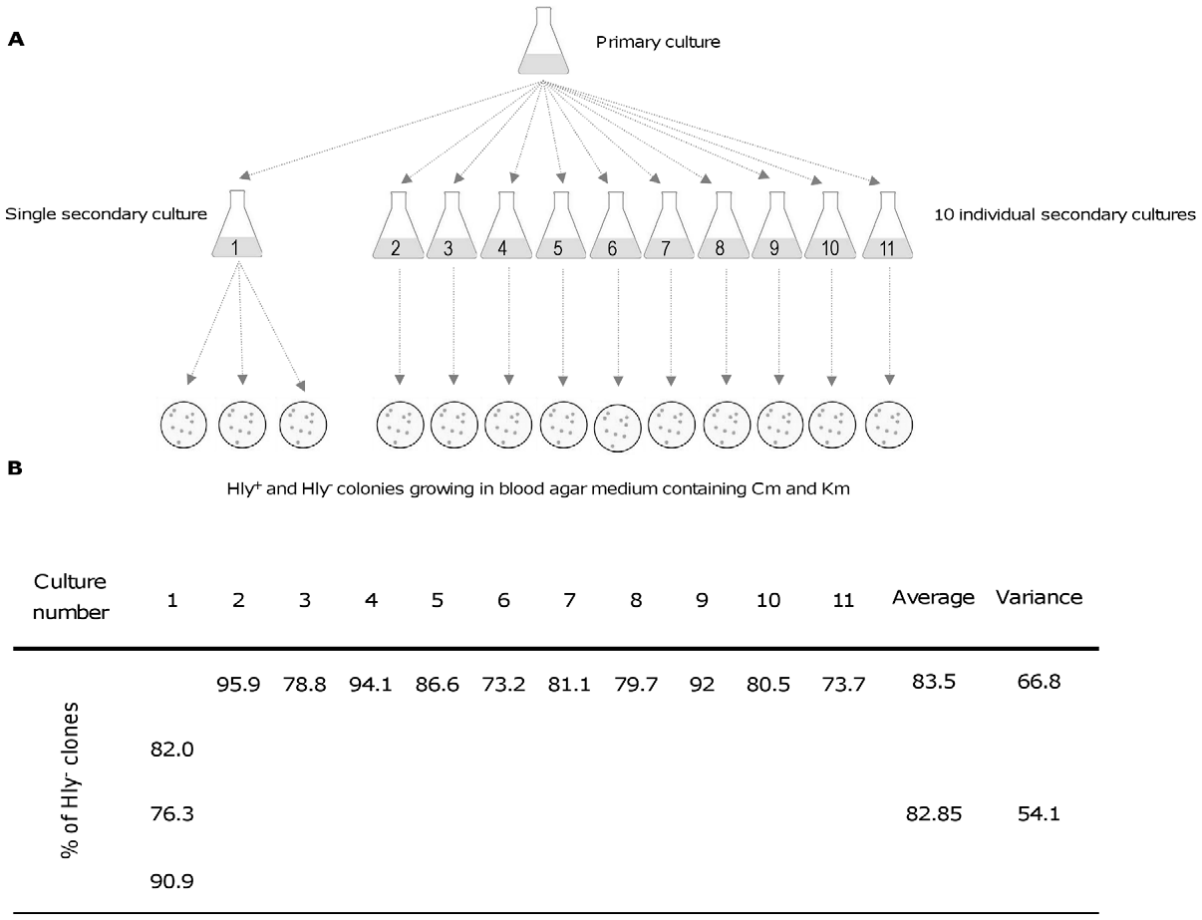
Fluctuation test. A. Shows the protocol used. The test was designed to determine if Hly^-^ clones arose prior to exposure to km or specifically in response to exposure. A primary culture of strain MG1655HY (pANN202-312R) was grown in LB medium containing cm but lacking km. Eleven secondary cultures were performed by transferring small amounts of the primary culture (final cell concentration in the secondary culture, 10^3^ cells/ml) into the same culture medium. These cultures underwent many rounds of cell division. From one of the cultures, three replicate subsamples were plated on blood agar plates containing cm and km. From the rest of the cultures, a single sample was plated onto identical plates. B. The percentage of Hly^-^ clones in the different viable counts on blood agar medium containing cm and km.

The fact that Hly^-^ clones are only detected when MG1655HY (pANN202-312R) cells are grown in LB medium containing km could be interpreted as either the presence of km in the culture directly accounts for the generation of non-hemolytic derivatives, or it just selects preexisting Hly^-^ clones because of their higher fitness in the presence of the antibiotic. To discern among these two possibilities, a fluctuation test was performed [28] (Figure 3). The mean and variance in the percentage of Hly^-^ clones on the blood agar plates was similar for replicates taken from a single secondary culture and for individual samples taken from different secondary cultures (10). Hence, the fluctuations observed were due to random sampling only, and not to the selection of a preexisting population of Hly^-^ clones. If this latter hypothesis should have been the case, significant fluctuations in the percentage of Hly^-^ clones in the ten independent cultures should have been observed, with a consequently drastic increase in the variance when compared to that of replicates from the same secondary culture. The fluctuation test clearly indicates that the presence of the antibiotic in the culture medium is the underlying cause of deletion of the *hly* operon and does not account for the selection of preexisting spontaneous deletions.

### The role of RecA, RpoS and the SOS response in generating Hly^-^ clones in the strain MG1655HY (pANN202-312R)

**Figure 4:**
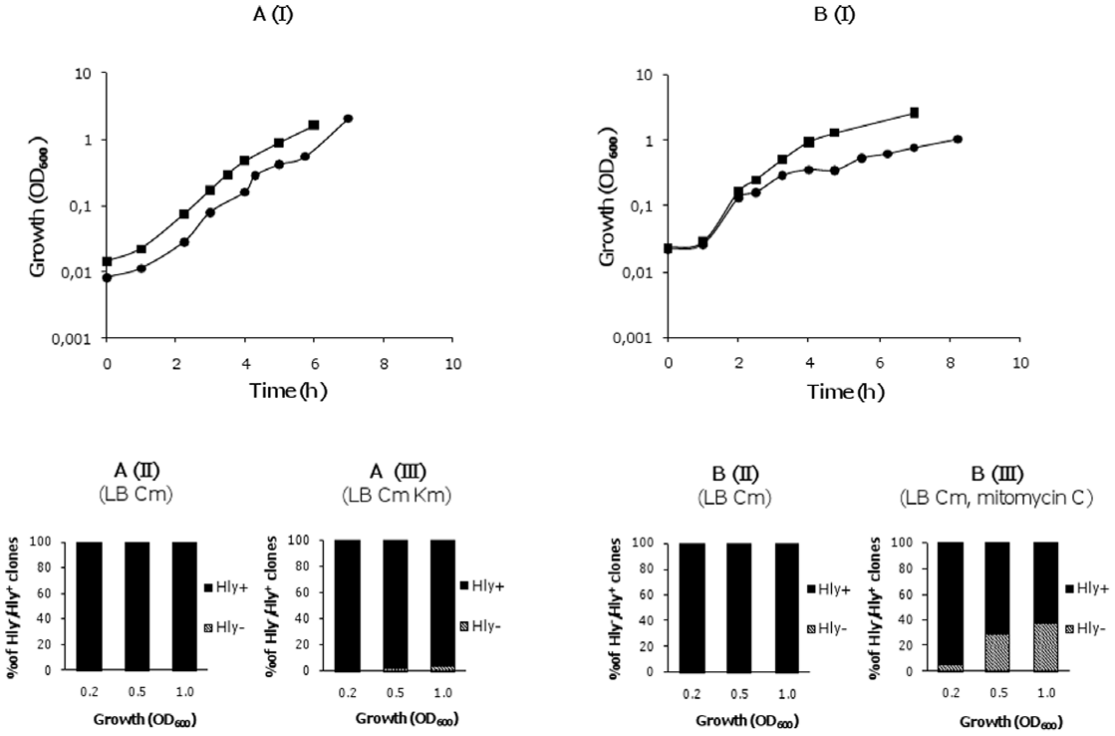
RecA protein and the SOS system play a role in the generation of Hly^-^ derivatives. A. I. Growth curves of the strain MG1655HY*recA* (pANN202-312R) in the absence (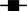) and presence (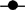) of km; II and III, the proportion of hemolytic and non-hemolytic clones in both cultures at different stages of the growth curve. B. I. Growth curves of the strain MG1655HY (pANN202-312R) in the absence (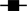) and presence (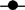) of mitomycin C. II and III, the proportion of hemolytic and non-hemolytic clones in both cultures at different stages of the growth curve.

We tested the dependence of the deletions leading to the Hly^-^ phenotype on the RecA protein. A *recA* mutant of strain MG1655HY was constructed by following the Datsenko&Wanner protocol (see Materials and Methods). *recA* knockout was confirmed both by PCR and by evidencing that P1-mediated transduction of different markers from strain MG1655 was not possible (data not shown). The strain MG1655HY*recA* was transformed with the plasmid pANN202-312R and the transformants grown in LB medium with and without km. Growth of strain MG1655HY*recA* (pANN202-312R) was monitored and the proportion of Hly^-^ clones was determined (Figure 4A). km significantly affected the growth rate of strain MG1655HY*recA* (pANN202-312R) and Hly^-^ derivatives could be isolated, but at a lower frequency than in the MG1655HY strain. Restriction analysis of plasmid DNA isolated from Hly^-^ clones derived from the *recA* strain indicated the presence of the two types of deletions previously identified (see Figure 2A). Hence, RecA function appears to facilitate recombination processes between the homologous IS*91* sequences, but deletions can also occur in the absence of this protein.

RpoS has been shown as a requirement for stationary-phase mutation and DNA amplification [19]. We assessed if deletion of the *hly* operon required RpoS function. To test this, an *rpoS* deletion mutant from the strain MG1655HY was constructed and the effect of km on the growth rate and generation of Hly^-^ derivatives was studied. The growth rate of the strain MG1655HY*rpoS* (pANN202-312R) was also reduced by the presence of km, and Hly^-^ clones harboring the previously characterized deletions were also detected (data not shown).

A well-characterized effect of several bactericidal antibiotics, including km, is the generation of highly deleterious hydroxyl radicals, which leads, among other responses, to the induction of the SOS response [29]. We decided to test if the SOS inducer mitomycin C might also account for the generation of Hly^-^ clones in cultures of the strain MG1655HY (pANN202-312R) in LB medium containing cm. Mitomycin C altered the growth rate and Hly^-^ derivatives could also be isolated (Figure 4B).

### A combination of a high kanamycin concentration (25 mg/l) and a high-level of hemolysin production are required to generate non-hemolytic derivatives from the strain MG1655HY

**Figure 5:**
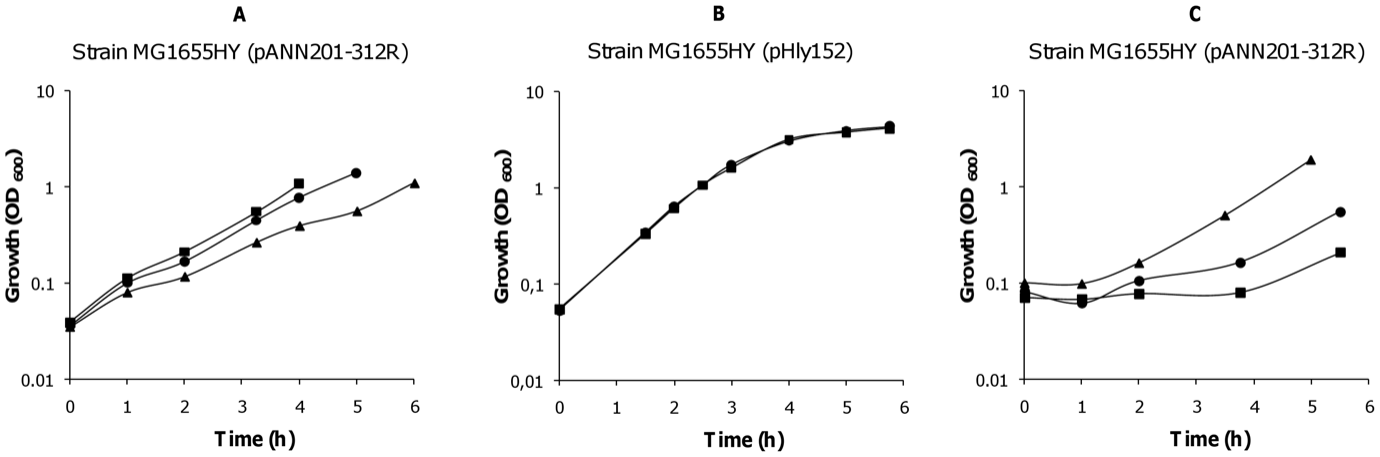
Effect of km concentration, copy number of the hemolytic plasmid and growth temperature on growth rate and generation of Hly^-^ clones. A. Growth curves of the strain MG1655HY (pANN202-312R) in LB medium containing either no km (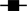), 12.5 mg/l km (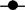) or 25 mg/l km (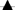). B. Growth curves of the strain MG1655HY (pHly152) in LB medium containing either no km (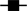) or 25 mg/l km (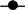). C. Growth curves of strain MG1655HY (pANN202-312R) in LB medium at 25 (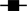), 30 (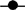) or 37°C (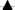). Hly^-^ derivatives were only detected in the culture of strain MG1655HY (pANN202-312R) grown in LB medium containing 25 mg/l km.

To gain insight into the mechanism underlying km-dependent deletion of the hemolytic determinant of plasmid pANN202-312R, we tested if the concentration of km in the culture medium influenced the generation of Hly^-^ clones. Strain MG1655HY (pANN202-312R) was grown in LB medium with cm (50 mg/l) and different concentrations of km (0, 12.5 and 25 mg/l). Growth and generation of Hly^-^ clones were monitored (Figure 5A). Remarkably, when the km concentration was reduced to 12.5 mg/l the growth rate was similar to that obtained in LB medium and the generation of Hly^-^ clones was no longer observed.

Next we studied if the copy number of the *hly* operon would influence the sensitivity to 25 mg/l of km and the generation of Hly^-^ derivatives. Strain MG1655HY harboring the parental low-copy number plasmid pHly152 was grown in LB medium containing no km and LB medium supplemented with 25 mg/l of km (Figure 5B). When growing in the presence of a km concentration of 25 mg/l, no Hly^-^ derivatives were obtained from strain MG1655HY (pHly152). Hence, if hemolysin production is decreased by reducing the copy number of the hemolytic plasmid, no Hly^-^ derivatives are obtained in LB medium containing 25 mg/l of km.

We also determined if plasmids pHly152 or pANN202-312R would modify the sensitivity to km of strain MG1655HY. The minimal inhibitory concentration (MIC) was determined. When comparing the km sensitivity of strains MG1655HY and MG1655HY (pHly152) no significant differences were observed. The presence of the plasmid did not alter the MIC value (data not shown). However, the km MIC was determined for the strains MG1655HY, MG1655HY (pANN202-312R) and MG1655HY (pANN202-312R' (Δ*hly*)), and a very important reduction (10 fold) in the MIC was observed when the strain carries the plasmid pANN202-312R (1000 µg/ml versus 100 µg/ml). Nevertheless, cells harboring plasmid pANN202-312R' exhibited a km MIC similar to that of plasmid-free cells.

Taking into account that km (25 mg/l) influences the growth rate of strain MG1655HY (pANN202-312R), we decided to rule out that the generation of Hly^-^ clones is a consequence of an alteration in the growth rate. Strain MG1655HY (pANN202-312R) was grown in LB medium containing cm (50 mg/l) and no km, at 37, 30 and at 25°C. Growth at 25°C resulted in a significant reduction in the growth rate (Figure 5C) but no Hly^-^ derivatives could be observed in the absence of km.

The above reported results suggest that generation of Hly^-^ derivatives takes place in the hemolysin overproducing strain MG1655HY (pANN202-312R) when it grows in medium containing a km concentration of at least 25 mg/l. Whereas that km concentration is not bactericidal for these cells (km MIC is 100 mg/l), it causes significant effects on the growth rate and also accounts for the generation of Hly^-^ derivatives.

### Subinhibitory concentrations of km and other antibiotics result in the deletion of the hemolysin operon in the wild-type MG1655 (pHly152) strain

The biochemical basis underlying the generation of Hly^-^ derivatives in MG1655HY (pANN202-312R) cells growing in medium containing 25 mg/l of km remains to be determined. Nevertheless, the studies described above suggest that 25 mg/l of km is subinhibitory in the strain MG1655HY (pANN202-312R). Growth in the presence of 12.5 mg/l km results both in a significant increase in the growth rate and in the absence of Hly^-^ clones. A feasible explanation for the high sensitivity to km showed by the strain MG1655HY (pANN202-312R) relies on the fact that the ATP-demanding process of producing such high levels of hemolysin would render cells inefficient in completely phosphorylating (inactivating) km at this concentration.

**Figure 6:**
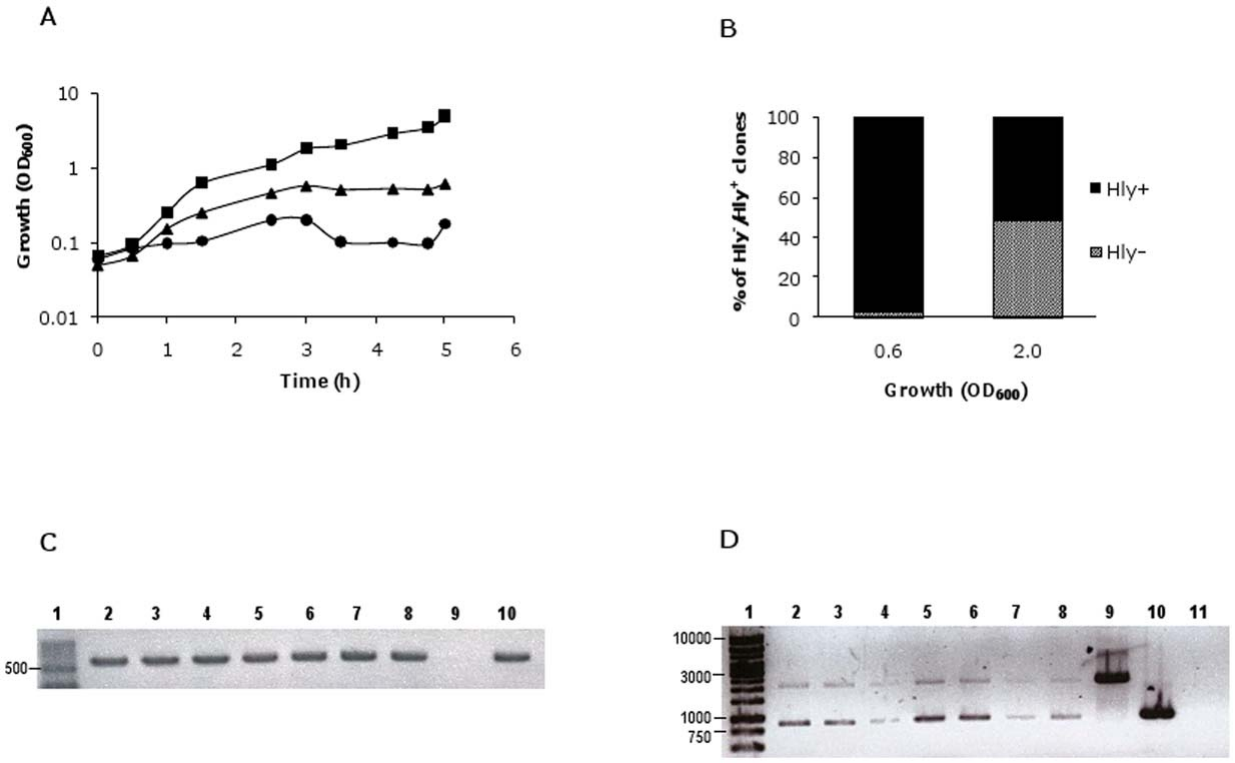
Subinhibitory km concentrations result in deletion of Hly sequences in strain MG1655 (pHly152). A. Growth curves of strain MG1655 (pHly152) in LB medium containing no km (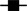), 5 (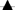) and 10 (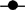) mg/l of km. B. The percentage of Hly^-^ clones in the culture grown in LB km (5 mg/l). C. PCR analysis using primers PANR8 and NBM18 to confirm the presence of pHly152 in seven Hly^-^ clones (lanes 2 to 8). Lane 1, molecular mass marker. Lane 9, negative control (strain MG1655). Lane 10, positive control (strain MG1655 (pHly152)). D. PCR analysis to confirm deletions 1 and 2 in the Hly^-^ clones. Primers used were PANR1 and PANR6, and the fragments amplified were of 875 bp and 2126 bp for the deletions 1 and 2 respectively.

Assuming as correct the hypothesis that a km concentration of 25 mg/l is subinhibitory for strain MG1655HY (pANN202-312R) and that this results in the stress-induced deletion of the *hly* sequences, it should be expected that the deletion of the *hly* operon could be also detected by growing the wt strain MG1655 harboring the parental low-copy number hemolytic plasmid pHly152 in the presence of km concentrations that result inhibitory for an *E. coli* strain lacking a km^R^ determinant. To assess this, the strain MG1655 (pHly152) was grown in LB medium containing 0, 1, 2, 5 and 10 mg/l of km (Figure 6A). A km concentration of 5 mg/l was the highest antibiotic concentration that allowed cells to grow. Thus, cells growing in liquid LB medium containing 5 mg/l of km were plated on blood agar at different stages of the growth curve. Several non-hemolytic colonies could be detected (48% at an OD_600_ of 2.0, Figure 6B). The Hly^-^ phenotype could be due either to plasmid curing or to the deletion of *hly* sequences. To distinguish between these cases, a PCR analysis of several of the Hly^-^ clones was performed using the primers PANR8 and NBM18 (Table 1, Figure 6C). The presence of the hemolytic plasmid was observed in several independent experiments. To determine which deletion of the *hly* sequences had taken place, different Hly^-^ clones were tested with primer pairs PANR1 and PANR6 (Table 1, Figure 2B). It was observed that both previously described types of deletion occurred, even within the same colony (Figure 6D). These results verify that subinhibitory km concentrations significantly increase the deletion rate of the *hly* operon of the plasmid pHly152.

**Table 1:** Oligonucleotides used in this study.

Name	Sequence (5′ 3′)	Purpose
PANR8	TCTGCGTGGAAGTATGAGC	To determine the presence/absence of pHly152
NBM18	CCATGCTGATGTGGCGCTTA	
PANR1	GATGATGCCACAAAATGGAT	To determine which recombination took place
PANR6	TTCCTGACACAGAACCGTAA	
NBM1	ACTCAGGAAACCGTAGTACCT	To sequence pANN202-312R
NBM3	GTAAAAGCCGCGAGGACAACG	
NBM4	GAAGCCAAGTCAAACAACAG	
NBM5	GCTGCTTCCTTCAACTGCCA	
NBM6	GGATTGCGCACCGGAAACCC	
NBM8	GATGCTTCCCCGTTTTGCCG	
NBM12	TATGACGCCACCCTACCAGT	
NBM14	CATTGCCATTGAAGCGGAGC	
RECAH1	CAGAACATATTGACTATCCGGTATTACCCGGCATGACAGGAGTAAAAATGGTGTAGGCTGGAGCTGCTTC	To construct *recA* mutants
RECAH2	ATGCGACCCTTGTGTTACAAACAAGACGATTAAAAATTTCGTTAGTTTCCATATGAATATCCTCCTTAGT	
RECAUP	CTTGTGGCAACAATTTCTACA	To check for *recA* mutation
RECADOWN	TCATGGCATATCCTTACAACT	
RPOSUP	TGCCGCAGCGATAAATCG	To check for *rpoS* mutation
RPOSDOWN	CGGACCTTTTATTGTGCACAG	

**Figure 7:**
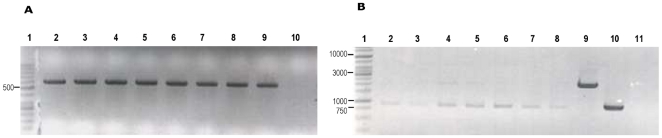
Deletion of the *hly* operon in strain MG1655 (pHly152) growing with a subinhibitory ampicillin concentration. Analysis of seven Hly^-^ clones isolated from strain MG1655 (pHly152) grown in LB medium containing ap (10 mg/l). A. PCR analysis using primers PANR8 and NBM18 to confirm the presence of pHly152. B. PCR analysis to confirm deletions 1 and 2 in the Hly^-^ clones. Primers used were PANR1 and PANR6, and the fragments amplified were of 875 bp and 2126 bp for the deletions 1 and 2 respectively.

Finally, the effect of subinhibitory concentrations of other antibiotics such as ampicillin (ap) was also tested. In this case the maximal antibiotic concentration allowing MG1655 (pHly152) cells to grow was of 10 mg/l. Again, Hly^-^ clones could readily be detected when cells were grown in LB ap (10 mg/l) medium, and those harboring the plasmid pHly152 exhibited the previously characterized deletions of the *hly* genes (Figure 7).

## Discussion

Several studies about the effect of antibiotics on bacterial cells have been performed using antibiotic concentrations known to either kill or inhibit the growth of their target cells. The knowledge of the effect on bacteria of subinhibitory antibiotic concentrations has been significantly enhanced in the last years (as reviewed by Davies *et al.* [30] and Couce and Blazquez [31]). Among other effects, exposure to subinhibitory concentrations of antibiotics leads to increased mutagenesis rates [31], [32], alterations in the global transcriptional pattern [33], [34], lateral gene transfer [35] and intrachromosomal recombination [36], [37]. In this report we provide experimental data that further support the relationship between antibiotics and bacterial genetic variability. Our interest in studying an unexpected observation led us to demonstrate that exposure to subinhibitory concentrations of antibiotics may result in genomic rearrangements that significantly modify the bacterial phenotype. As stated by Shapiro [38], serendipity has led to a more complete view of the capacity that the bacterial cells exhibit for restructuring their genomes in response to several stimuli.

IS*91* was first characterized in α-hemolytic plasmids of *E. coli* [6], [39]. Presently IS*91* is the prototype of an expanding family of insertion elements (the IS*CR* elements, [40]) that have been found adjacent to both virulence determinants ([7], [12], [13], [41]) and antibiotic resistance genes [40]. Remarkably, the IS*CR* family is widespread among bacteria, but IS*91* elements are mainly restricted to *E. coli* and *Shigella* strains. In both *E. coli* and *Shigella* IS*91* has previously been associated to genetic rearrangements. The presence of either complete or partial IS*91* sequences flanking the genes encoding heat-labile enterotoxins (*eltAB*) of enterotoxigenic *E. coli* strains resulted, among other effects, in spontaneous transpositions of the *eltAB* genes to the target plasmid pSU2600 [41]. In *S. flexneri* strain 2a, recombination between two flanking IS*91* elements underlies one of the three mechanisms that result in deletion of the SLR pathogenicity island [21]. Our results are in accordance with these data. Remarkably, the 17,010 bp *Sal*I/*Sal*I DNA fragment that includes the Hly operon of plasmid pHly152 cloned in pACYC184 contains only incomplete IS*91* copies and, in fact, some of these sequences, flanking the *hly* operon in a directed repeat orientation, are responsible for the recombinational events that lead to the deletion of two different DNA fragments. In some circumstances, RecA-dependent homologous recombination may be the mechanism underlying deletion of the *hly* genes (e.g. when the SOS system is induced), but RecA-independent recombination also takes place. RecA-independent IS*91*-mediated genetic rearrangements were also described for the *eltAB* genes of enterotoxigenic *E. coli* strains [41]. The mechanism that accounts for the deletion of the *hly* operon from plasmid pHly152 is necessarily independent of the IS*91* TnpA transposase, since no complete copies of the *tnpA* gene are present either in the plasmid pANN202-312R or in the chromosome of the *E. coli* strain MG1655 (our unpublished results). We hypothesized that ORF121, the second and yet uncharacterized ORF encoded by IS*91*, which is present in one of the partial IS*91* copies of the plasmid pANN202-312R, might play a role in these recombinational processes. However, deletion of that sequence did not influence loss of the hemolysin operon in the strain MG1655HY (pANN202-312R) (our unpublished results). Replication is a key process involved in RecA-independent instability of repetitive DNA sequences [42]. Hence, antibiotic-mediated alterations in DNA replication might account for deletion of the *hly* sequences in the absence of RecA.

Genomic analysis data reveal that IS*91* is predominantly present in plasmids isolated from enterohemorragic *E. coli* strains (serotypes O111, O26 and O157), or in the chromosome and/or plasmids from *Shigella* isolates (our unpublished results). Interestingly, the six IS*91* copies present in the chromosome of the *S. flexneri* strain 2457T cluster together and flank several virulence determinants [43]. Local hopping was proposed to explain the clustering pattern of these determinants. Nevertheless, if these IS*91* elements can under certain circumstances be responsible for genomic rearrangements, then their random distribution in the *Shigella* chromosome would in many cases result in lethality. The fact that IS*91* flanked virulence determinants are restricted to certain *E. coli*/*Shigella* strains may indicate that these strains display highly variable pathogenic phenotypes. Therefore, strains that do not incorporate IS*91*-like elements prevent genomic instability due to the presence of these elements in their genomes.

Several antibiotics such as trimethoprim, quinolones and β-lactams induce the SOS response [44]. It has recently been shown that induction of the SOS response can lead to integrons recombination and the expression of antibiotic resistance determinants in *E. coli* and *Vibrio cholerae* [44]. In *Staphylococcus aureus*, induction of the SOS response leads to dissemination of pathogenicity islands that are packaged in bacteriophages [45]. In this paper we present further evidence demonstrating that induction of the SOS response underlies the excision of a virulence determinant in *E. coli*. Hence, factors resulting in SOS induction (among them, several antibiotics) may also account for different types of genomic rearrangements in bacteria.

It is likely that bacteria in natural environments encounter subinhibitory concentrations of antibiotics more often than inhibitory concentrations. Moreover, clinical situations where bacteria may be exposed to low levels of antibiotics can occur due to several reasons (e.g. incomplete antibiotic treatment of an infection or the differential pharmacological response of certain tissues to antibiotics). In the case of the *E. coli*/*Shigella* pathogenic strains that harbor virulence determinants flanked by IS*91* elements, exposure to low levels of antibiotics may result in genetic rearrangements and the deletion of IS*91*-flanked determinants. Whereas some changes might be deleterious, others may confer new phenotypes that potentiate their virulence in specific niches, hence increasing their invasiveness. Therefore, noncompliance with or incomplete antibiotic treatments should be avoided. Furthermore, a better understanding of the mechanisms underlying IS*91*-mediated deletion of virulence determinants in response to antibiotic exposure may help to better define additional targets for antimicrobial therapy.

## Materials and Methods

### Bacterial strains, plasmids, and media

The bacterial strains and plasmids used in this study are listed in Table 2. The plasmid pANN202-312R carries the *hly*CABD gene cluster from the low-copy-number hemolytic plasmid pHly152, and was previously described by Godessart *et al.* [27]. *E. coli* strains were grown on Luria–Bertani (LB) medium (10 g NaCl, 10 g tryptone and 5 g yeast extract per liter) at 37°C under aerobic conditions. Blood agar was LB medium supplemented with a 5% of sheep blood defibrinated (Oxoid). In the growth medium the following antibiotics were used: chloramphenicol (cm), 50 µg/ml; kanamycin (km), 25 µg/ml (unless otherwise stated) and ampicillin (ap), 10 µg/ml. To study generation of Hly^-^ derivatives from strain MG1655HY as well as from its *recA* and *rpoS* derivatives, the corresponding strain was transformed first with plasmid pANN202-312R. Transformant colonies were selected in blood agar containing cm. Single colonies were used to inoculate LB-cm cultures that were grown overnight and used to inoculate (1∶100) erlenmeyer flasks containing LB cm and LB cm km. Five independent colonies from five independent transformation experiments were studied.

**Table 2:** Strains and plasmids used in this study.

Strain or plasmid	Description	Source or reference
***E. coli*** ** K-12 laboratory strains:**		
MG1655	F- lambda- *ilvG*- *rfb*-50 *rph*-1	[50]
BSN26H	MC4100 *trp*::Tn10 *hha*::Tn5	[47]
BSN26Y	MC4100 *trp*::Tn10 Δ*ydgT*	[25]
MG1655HY	F- lambda- *ilvG*- *rfb*-50 *rph*-1 *hha ydgT*	This work
MG1655HYΔ*recA*	F- lambda- *ilvG*- *rfb*-50 *rph*-1 *hha ydgT recA*	This work
MG1655HYΔ*rpoS*	F- lambda- *ilvG*- *rfb*-50 *rph*-1 *hha ydgT rpoS*	This work
RH90	MC4100, *rpoS*359::Tn10, Tc^R^	[48]
**Plasmids:**		
pHly152	*hlyR hlyCABD*	[51]
pANN202-321R	*hlyR hlyCABD* cloned in pACYC184, cm^r^	[27]
pANN202-321R'	pANN202-321R Δ*hly*	This work

### Molecular techniques

Plasmid DNA preparations were carried out from 5 ml of overnight cultures according to the Qiagen kit protocol. Restriction analysis of the plasmid pANN202-312R isolated from hemolytic and non-hemolytic colonies was performed by standard techniques. PCR amplifications of the plasmid pHly152 were performed with a MJ Mini Gradient Thermal Cycler (BioRad®) using the oligonucleotides PANR1-PANR6 and PANR8-NBM18 (listed in table 1) at a Tm of 58 and 56°C respectively.

### DNA sequencing

Sequencing was performed with the ABI PRISM Big Dye II Terminator Cycle Sequencing Ready Reaction Kit (Applied Biosystems) in an ABI PRISM 3700 DNA Analyzer (Applied Biosystems), according to the manufacturer's instructions. The sequencing primers used are listed in Table 1.

### Genetic manipulations

P1*vir* mediated transduction was performed as previously described (Miller, [46]) to construct MG1655*hha ydgT* (HY) using lysates obtained from the strains BSN26*hha* [47] and BSN26*ydgT* [25]. An MG1655HY*rpoS* mutant was obtained by P1*vir* transduction using a lysate from the RH90 strain [48]. The P1*vir* mediated transduction was also used to check the *recA* deletion. A *recA* deletion mutant was obtained by one-step inactivation using PCR products, as previously described [49]. We used the sequence of the *recA* gene to define the corresponding amplification primers. The antibiotic resistance of the plasmid pKD3 (cm) was amplified using primers RECAH1 and RECAH2 (Table 1), corresponding to sequences P1 and P2 of plasmid pKD3, with homology extensions corresponding to nucleotides 2821789-2821833, and 2820701-2820752 of the complete genome of *E. coli* K-12 MG1655 (NC_000913). The PCR product was *Dpn*I digested, purified and used to electroporate strain MG1655HY carrying the plasmid pKD46 grown at 30°C in the presence of arabinose 10 mM, conditions where the expression of Red recombinase was induced. Recombinants were selected at 37°C in LB medium containing chloramphenicol (cm) and then tested for the presence of pKD46. All the recombinants obtained were checked with RECAUP and RECADOWN primers (Table 1) and only one was found correct. We termed this mutant MG1655HY*recA*.

### Determination of hemolysin production

Strains carrying hemolytic plasmids (pANN202-312R or pHly152) were grown in presence and in absence of the antibiotic km. We checked hemolysin production by visualizing hemolysin haloes on blood agar plates.

### Fluctuation test (Luria–Delbrück experiment)

An overnight culture of the strain MG1655HY (pANN202-312R) was used to inoculate eleven erlenmeyer flasks containing 20 ml of LB cm medium (final cell concentration after inoculation, 10^3^ cells/ml). Cultures were incubated at 37°C to a final OD_600_ = 1. Serial dilutions were then made. From one of the subcultures, three independent serial dilutions were made. From the other ten cultures only one serial dilution was made. Appropriate dilutions were then plated on blood agar containing cm and km and the proportion of Hly^-^ clones was determined.

## Supporting Information

Figure S1 **Location within the complete IS** ***91*** **sequence of the five partial IS** ***91*** **sequences present in plasmids pANN202-312R and pHly152.**
(TIF)
