# Supplementary figures and images for: Antibiotics Shaping Bacterial Genome: Deletion of an IS91 Flanked Virulence Determinant upon Exposure to Subinhibitory Antibiotic Concentrations
Source: PLoS One. 2011 Nov 11;6(11):e27606. doi: 10.1371/journal.pone.0027606 (PMC3214074; doi:10.1371/journal.pone.0027606)

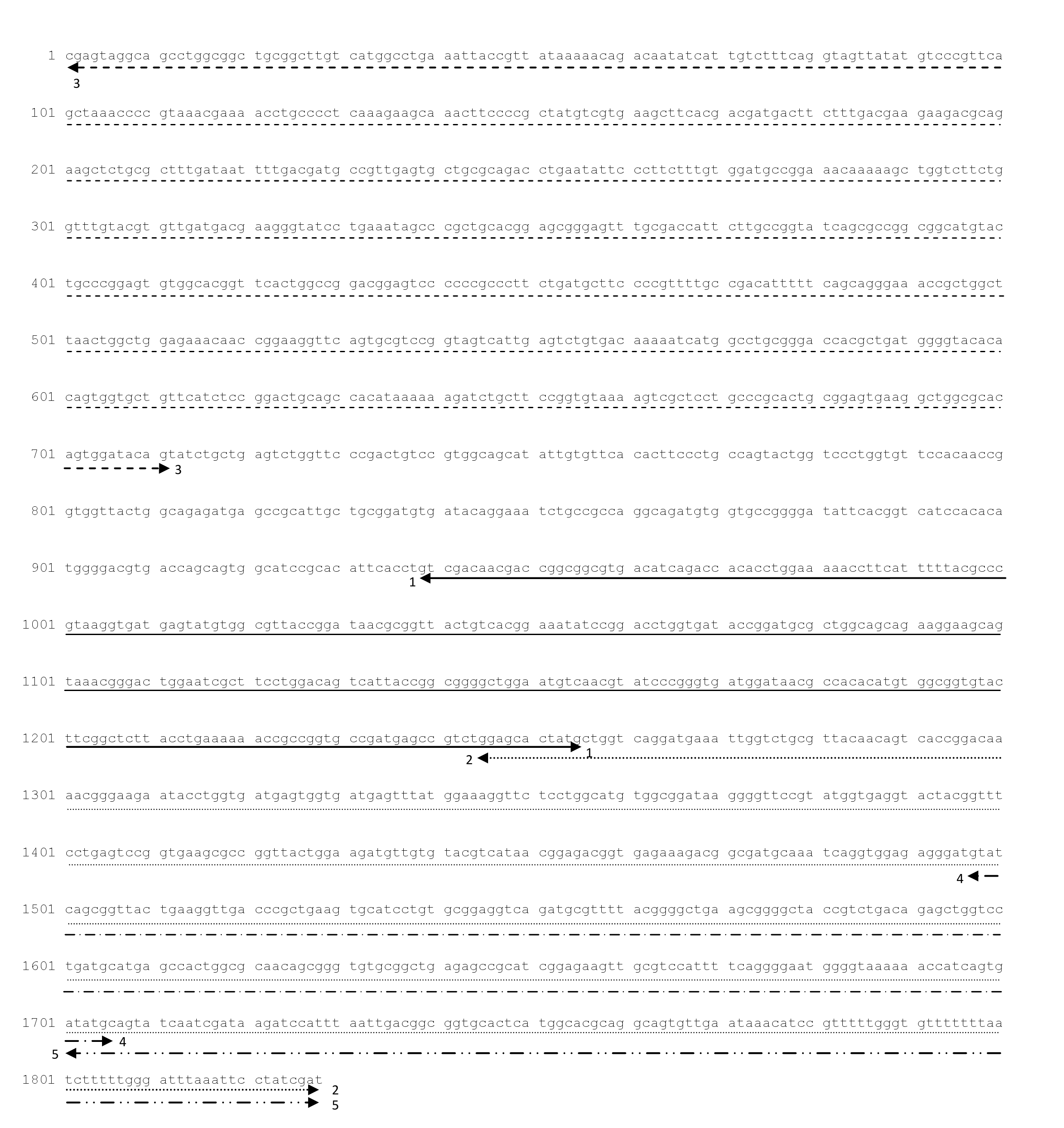

Supplement: Figure S1 — Location within the complete IS 91 sequence of the five partial IS 91 sequences present in plasmids pANN202-312R and pHly152. (TIF) [file pone.0027606.s001.tif]
